# Telehealth-Readiness, Healthcare Access, and Cardiovascular Health in the Deep South: A Spatial Perspective

**DOI:** 10.3390/ijerph22071020

**Published:** 2025-06-27

**Authors:** Ruaa Al Juboori, Dylan Barker, Andrew Yockey, Elizabeth Swindell, Riley Morgan, Neva Agarwala

**Affiliations:** 1 Department of Public Health, School of Applied Sciences, The University of Mississippi, University, MS 38677, USA; dabarke1@go.olemiss.edu (D.B.); rayocke1@olemiss.edu (A.Y.); ecswinde@go.olemiss.edu (E.S.); jrmorga4@go.olemiss.edu (R.M.); 2 Department of Health Science, South College, 2600 Century Parkway NE, Atlanta, GA 30345, USA; nagarwala@south.edu

**Keywords:** cardiovascular disease, telehealth readiness, spatial analysis, healthcare provider shortages, broadband access, Deep South, Moran’s I

## Abstract

Background: Cardiovascular disease remains a leading cause of preventable mortality in the United States, with rural counties in the Deep South experiencing disproportionately high burdens. Grounded in the Andersen healthcare utilization model, this study examined how enabling resources, predisposing characteristics, and access-related barriers relate to coronary heart disease (CHD) prevalence and mortality. Methods: This ecological analysis included 418 counties across Alabama, Georgia, Louisiana, Mississippi, and South Carolina. Using Local Indicators of Spatial Association (LISA) and multivariable linear regression, we tested three theory-based hypotheses and assessed the spatial clustering of CHD outcomes, while identifying key structural and sociodemographic predictors. Results: Counties with greater rurality and fewer healthcare providers exhibited significantly higher rates of CHD prevalence and mortality. Primary care provider availability and higher household income were protective factors. Digital exclusion, measured by lack of access to computers or mobile devices, was significantly associated with higher CHD prevalence and mortality. Spatial analysis identified the counties with better-than-expected cardiovascular outcomes despite structural disadvantages, suggesting the potential role of localized resilience factors and unmeasured community-level interventions. Conclusions: The findings affirm the relevance of the Andersen model for understanding rural health disparities and highlight the importance of investing in both digital infrastructure and healthcare capacity. Expanding telehealth without addressing provider shortages and social determinants may be insufficient. Local policy innovations and community resilience mechanisms may offer scalable models for improving cardiovascular health in disadvantaged areas.

## 1. Introduction

### 1.1. Background

Cardiovascular disease remains the leading cause of death in the United States, accounting for nearly 702,880 deaths in 2022 [1]. Yet, this burden is not distributed evenly. Rural areas in the Deep South consistently experience some of the highest cardiovascular mortality rates in the country. For example, Mississippi reported the highest heart disease mortality rate and the second-highest stroke mortality rate in the U.S. in 2017 [2]. This pattern has been described in the literature as the southern rural health penalty, in which rural Southern populations face disproportionately high rates of preventable conditions such as hypertension, diabetes, obesity, and cardiovascular disease [3].

These health disparities are shaped by systemic challenges including geographic isolation, under-resourced healthcare systems, and low educational and economic attainment [4]. Many counties in the Deep South experience significant shortages in both primary care providers and specialists, such as cardiologists and nurse practitioners [5]. At the same time, hospital closures and a lack of local preventive care services exacerbate the chronic underdiagnosis and undertreatment of cardiovascular disease [3,6]. Transportation barriers further hinder access, with many rural residents traveling long distances, often without reliable transit, to reach care.

Digital infrastructure plays an increasingly important role in reducing these barriers. Telehealth has demonstrated the potential to expand access to care in rural areas, particularly for the management of chronic diseases like heart failure and hypertension [5]. However, in many rural Deep South counties, poor broadband availability and high rates of households lacking computers or cell phones undermine these innovations. As a result, the communities most in need of remote care options are often the least equipped to use them.

While rural counties are often grouped together, they are not homogenous. Many face compounding disadvantages across infrastructure, socioeconomic status, and provider availability. Understanding the geographic distribution of these structural barriers is essential to designing healthcare interventions.

### 1.2. Literature Review

#### 1.2.1. Rural Health Conditions and Structural Barriers

A growing body of research has examined the health challenges faced by residents of rural areas in the United States, especially in the Deep South [7,8,9,10,11,12]. These challenges are shaped based on limited healthcare provider availability, historical underinvestment, geographic isolation, and persistent socioeconomic hardship [10,13,14]. Counties across Mississippi, Alabama, and Louisiana often share a concentration of risk factors like aging populations, a high prevalence of chronic disease, and limited public health infrastructure that place them at greater risk for adverse health outcomes when compared to more urban areas [7,15].

Rural residents frequently encounter long travel distances to care, a lack of reliable transportation, and a lack of service for preventive or specialty care [16,17]. Additionally, many of these counties have seen reductions in healthcare access due to hospital closures and constrained clinical capacity, often linked to broader financial pressures facing rural health systems [18]. These combined conditions make timely diagnosis and consistent disease management more difficult, particularly for chronic conditions like CHD (coronary heart disease). Despite increasing recognition of these challenges, few studies have used geospatial approaches to explore how place-based factors are associated with the geographic distribution of poor cardiovascular outcomes. Understanding where service limitations and resource gaps are most concentrated can help inform more targeted health interventions.

#### 1.2.2. Digital Infrastructure as a Social Determinant of Health

In recent years, digital inclusion, defined by access to broadband internet, digital devices, and technological literacy, has emerged as one of the social determinants of health. The National Digital Inclusion Alliance identifies lack of broadband access as a driver of “digital redlining,” which disproportionately affects rural and low-income communities [19]. Research shows that digital exclusion is strongly correlated with reduced access to telehealth, lower patient engagement, and diminished health service utilization, particularly in rural regions [20,21].

According to the Federal Communications Commission (FCC)’s 2024 Broadband Deployment Report, approximately 22.4% of rural Americans lack access to fixed terrestrial broadband with speeds of 25/3 Mbps, compared to only 1.5% of urban residents [22]. This urban–rural gap is particularly pronounced in states like Mississippi and Louisiana, where rural broadband deployment continues to lag behind national benchmarks [22]. This digital divide directly impedes the standard component of care including the scalability of telehealth interventions and limits access to electronic health records, patient portals, and remote consultations. Despite growing recognition, few studies have linked county-level digital infrastructure data with cardiovascular outcomes. Even fewer studies have used spatial analysis to examine how digital readiness aligns with provider access and socioeconomic factors to influence disease burden.

#### 1.2.3. Telehealth and Cardiovascular Outcomes

Telehealth is widely recognized as a promising tool to improve access to care in rural and medically underserved areas, particularly for chronic diseases such as hypertension, diabetes, and cardiovascular disease [23,24,25]. Evidence from health systems and pilot programs suggests that telehealth platforms can reduce hospital readmissions, improve medication adherence, and enhance disease management for patients with CHD [26,27,28].

However, the successful implementation of telehealth depends on both technological infrastructure and provider readiness. Studies have shown that counties with both low provider density and poor broadband access have substantially lower telehealth utilization [29,30]. In such regions, telehealth may not reach the populations it is designed to serve. This undermines its potential to reduce disparities. Furthermore, most telehealth studies use individual-or system-level data, this approach overlooks geographic differences in implementation and outcomes.

This study addresses several critical gaps. First, it applies a geospatial framework to assess how digital infrastructure, provider access, and sociodemographic conditions jointly shape cardiovascular outcomes across 418 counties in Alabama, Georgia, Louisiana, Mississippi, and South Carolina. Unlike prior research that treats rural areas as homogeneous, this study uses spatial clustering to identify the counties that outperform or underperform relative to risk. Second, it differentiates between broadband availability and household-level device access, an often overlooked but essential dimension of telehealth readiness. Finally, this study integrates structural indicators into a multivariable spatial analysis. This approach allows for the identification of modifiable, policy-relevant predictors to inform regional public health strategies.

### 1.3. Theoretical Framework

This study is guided by the Andersen healthcare utilization model, a framework for understanding the determinants of healthcare access and outcomes [31]. The model classifies the factors associated with healthcare utilization into three domains: predisposing characteristics, enabling resources, and need factors. These domains are suitable for this study, which investigates the structural and contextual predictors of CHD outcomes across counties in the Deep South. Predisposing characteristics refer to demographic characteristics that shape a population’s tendency to seek or engage with health services. In this study, the percentage of the population aged 65 and older is considered a predisposing factor, as older adults are more likely to require ongoing medical care and chronic disease management [32]. The racial composition of each county, captured through the percentage of non-Hispanic White residents, is also included as a predisposing factor, based on the literature indicating that race and ethnicity can shape patterns of healthcare use through cultural norms, historical barriers, and systemic differences in treatment access [33,34].

Enabling resources are those that facilitate or impede a population’s ability to obtain healthcare. Several variables in this study reflect enabling conditions. The density of primary care physicians, nurse practitioners, and cardiologists per county serves as a direct measure of healthcare system capacity, with lower availability linked to limited access to both preventive and specialized services. Digital infrastructure was also considered; the percentage of households without a computer or smartphone, along with broadband access levels, indicates telehealth readiness and technological capability. These indicators have become increasingly important for healthcare delivery, especially in rural settings where digital tools may substitute for in-person visits. Additionally, transportation access is included as an enabling factor, measured by the percentage of housing units without a vehicle. In areas where public transportation is limited or nonexistent, a lack of personal transportation can prevent residents from accessing routine or emergency care. Median household income, representing economic resources at the community level, is also considered an enabling factor, as higher-income counties may have more financial flexibility to support healthcare seeking behaviors.

Need factors represent the health conditions that drive demand for services. In this study, need is captured through two outcome variables: CHD prevalence and CHD mortality rate. CHD prevalence reflects the level of existing chronic illness in a population, while CHD mortality indicates the severity of outcomes and potentially unmet medical needs. These two measures together provide a comprehensive picture of the cardiovascular disease burden across counties. By applying the Andersen model, this study uses a theory-driven approach to organize and interpret the relationships between sociodemographic conditions, access-related infrastructure, and health outcomes.

### 1.4. Hypotheses

Building on the Andersen healthcare utilization model and the prior literature on rural healthcare access and cardiovascular disease, this study proposes three guiding hypotheses to guide the analysis and interpretation of findings.

**Hypothesis** **1.** *We hypothesize that counties with limited enabling resources, such as low healthcare provider availability, limited broadband infrastructure, and high levels of digital exclusion, will experience higher rates of CHD prevalence and mortality. This hypothesis is grounded in the assumption that both in-person care and telehealth services are essential for the prevention, diagnosis, and ongoing management of cardiovascular conditions. Inadequate digital or physical access to providers may result in delayed detection and poorer disease control.*

**Hypothesis** **2.** *We hypothesize that demographic and geographic predisposing factors, particularly higher proportions of older adults and higher levels of rurality, will be associated with an increased CHD burden. Older populations tend to have more chronic conditions, while rural settings are often characterized by geographic barriers to accessing health services. These predisposing factors may indirectly be associated with outcomes by shaping both the demand for and ease of accessing care.*

**Hypothesis** **3.** *We hypothesize that enabling resources such as greater provider density, higher broadband access, and higher household income will be protective factors associated with lower CHD prevalence and mortality. In contrast, higher percentages of uninsured individuals, households without a vehicle, and households lacking computers or smartphones are expected to be associated with worse outcomes. These conditions reduce the likelihood of engaging in preventive care and managing existing conditions effectively, particularly in contexts where telehealth could otherwise play a supportive role.*

## 2. Materials and Methods

### 2.1. Study Design and Data Sources

This ecological study examined the spatial distribution and social determinants of CHD and CHD-related mortality across counties in the Deep South states [35,36]. To test this study’s hypotheses, we used spatial clustering techniques and multivariable regression to assess how predisposing, enabling, and need-related factors influence cardiovascular outcomes. County-level data were obtained for 418 counties from publicly available sources, including health workforce statistics, socioeconomic indicators, and access-to-care metrics. The focus of the analysis was to identify geographic disparities and examine how rural social, demographic, and healthcare access variables are associated with the burden of CHD and CHD deaths. Table 1 provides detailed descriptions of the study variables used in the analysis.

### 2.2. Outcome Variables

Two primary dependent variables were analyzed: (1) CHD prevalence and (2) CHD deaths, with both measured as rates per county.

### 2.3. Independent Variables

A set of independent variables was selected to capture the relevant social, economic, demographic, and health service factors hypothesized to be associated with cardiovascular outcomes in rural settings. These variables are described in detail in Table 1.

### 2.4. Statistical Analysis

The analytical approach for this study was designed to align with both the research objectives and the theoretical foundation provided by the Andersen healthcare utilization model. Spatial analysis, specifically Local Indicators of Spatial Association (LISA) using Local Moran’s I, was employed to detect the statistically significant spatial clustering of CHD prevalence and mortality across counties. This method is widely used in public health to reveal regional hotspots or cold spots and identify patterns that may not be captured through traditional regression [40]. Queen contiguity spatial weights were applied to define neighboring county relationships [41]. This approach allows for the identification of high–high and low–low clusters.

Following spatial exploration, we used multivariable linear regression to estimate the associations between CHD outcomes and the enabling, predisposing, and contextual factors outlined in Table 1. Regression analysis is used in confirmatory research grounded in theoretical models like Andersen’s, as it allows for the testing of specific hypotheses about access to care and the structural predictors of health [31]. These techniques complement each other by combining geographic context with inferential modeling, offering a multidimensional understanding of cardiovascular disease burden in rural counties. These spatial analyses were performed using the rgeoda, sf, and tmap packages in R. All analyses were conducted using R version 4.4.2.

Regression models were constructed to test three a priori hypotheses derived from the Andersen healthcare utilization model: (1) that limited enabling resources (e.g., provider shortages, digital exclusion) would be associated with higher CHD prevalence and mortality; (2) that demographic and geographic predisposing factors (e.g., age, rurality) would correlate with CHD burden; and (3) that access-enabling conditions (e.g., broadband availability, income) would be protective. These hypotheses informed both model specification and variable selection.

The general form of the multivariable linear regression model used in this study is as follows:

*Yi* = *β*_0_ + *β*_1_*X*_1_*i* + *β*_2_*X*_2_*i* + … + *βkXki* + *εi* (1)

where *Yi* = the Outcome variable (CHD prevalence or CHD mortality) for county *i*;

*X*_1_*i* through *Xki* = the Independent variables for county *i* (as listed in Table 1);

*β*_0_ = the Intercept;

*β*_1_ through βk = the Estimated coefficients;

*εi* = the Error term.

Model assumptions were checked through residual plots and normality assessments, and multicollinearity was evaluated using variance inflation factors (VIFs). Only the variables with acceptable VIF values (<5) were retained in the final models to ensure model stability. The level of significance was set to be <0.05; regression Beta and the corresponding 95% CI were reported.

The analytic sample included 418 counties located in five Deep South states: Alabama, Georgia, Louisiana, Mississippi, and South Carolina. These counties were selected based on the availability of complete data on both outcome and independent variables. All data used in the analysis were aggregated at the county level, consistent with the ecological design of this study. No individual-level data were used, and all variables represent publicly available population-level indicators.

## 3. Results

The results are interpreted considering this study’s three guiding hypotheses, particularly regarding the role of enabling and predisposing factors on cardiovascular outcomes. The descriptive analysis revealed variability across Deep South counties in both healthcare access and enabling infrastructure. On average, 18.9% of the population was aged 65 or older (SD = 3.9%), with variation in insurance coverage (mean uninsured rate = 11.4%, SD = 3.6%) and access to technology; 10.3% of households lacked a computer/cell phone (SD = 4.8%), and 6.9% of households lacked a vehicle (SD = 3.3%). Those are two key indicators of infrastructure that could hinder the effectiveness of telehealth initiatives.

The mean rate of CHD prevalence was 8516.986 per 100,000 (SD = 1450.303), while the average CHD death rate was 447 per 100,000 (SD = 98.7). Healthcare resource availability, as measured by provider density, was low: the mean number of primary care physicians per resident was 0.04% (SD = 0.03%), with similarly sparse availability of nurse practitioners and cardiovascular disease specialists across counties. Please refer to Table 2 for a more detailed description of the study variables. 

### 3.1. Spatial Patterns

A LISA analysis, based on Local Moran’s I, revealed statistically significant spatial clustering for both CHD prevalence and CHD mortality across rural counties in the Deep South region. High–high clusters (areas with high values surrounded by similarly high neighbors) were consistently observed in parts of Mississippi, Alabama, and Louisiana, while low–low clusters appeared in southern Georgia, indicating regional disparities in cardiovascular burden. CHD prevalence clusters were particularly concentrated in the counties characterized by lower broadband access, high proportions of older adults, and higher rates of households lacking basic technology (computer/cell phone). These clusters also overlapped with areas that had low densities of primary care physicians and nurse practitioners. This highlights spatial gaps in preventive healthcare delivery.

**Figure 1:**
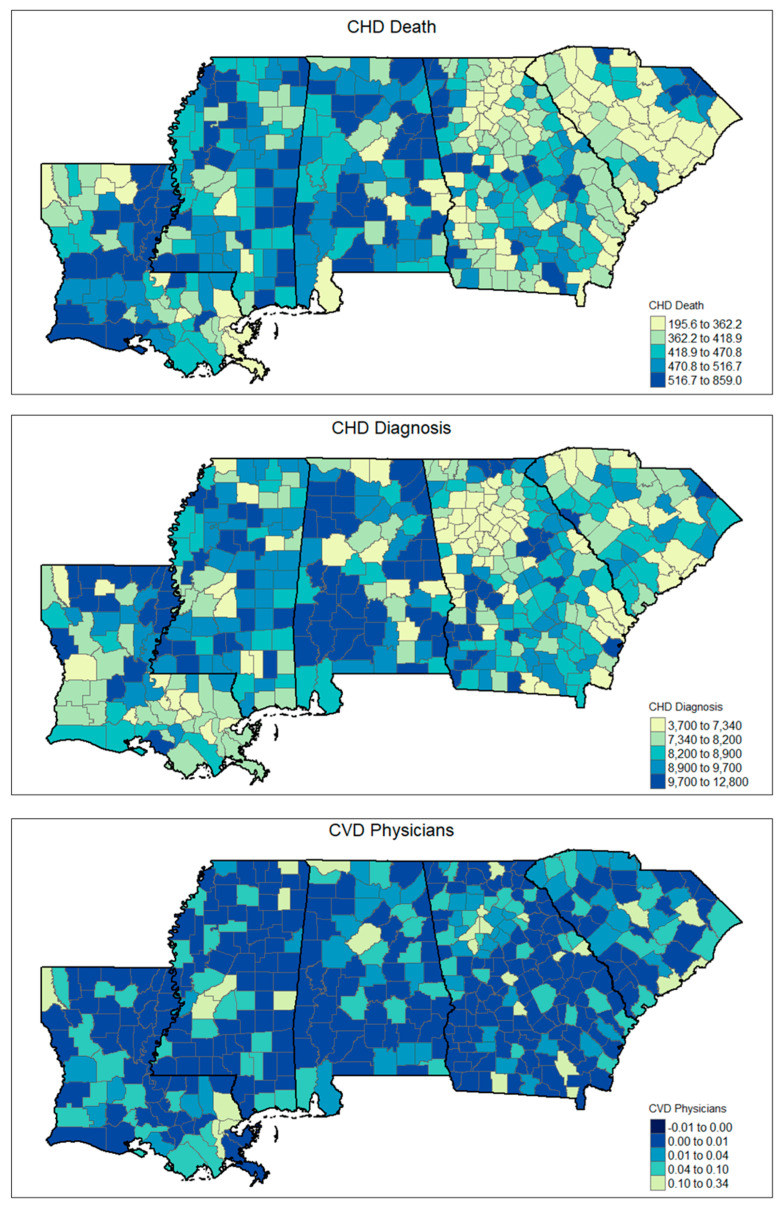

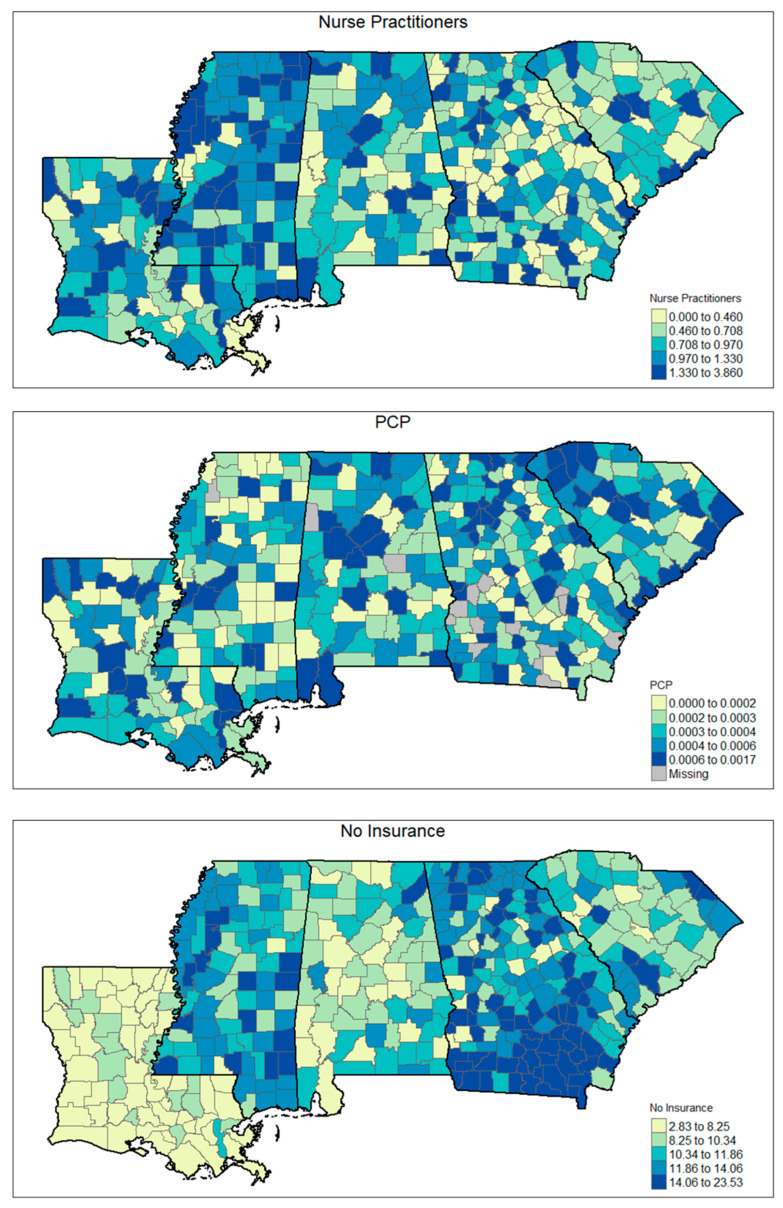

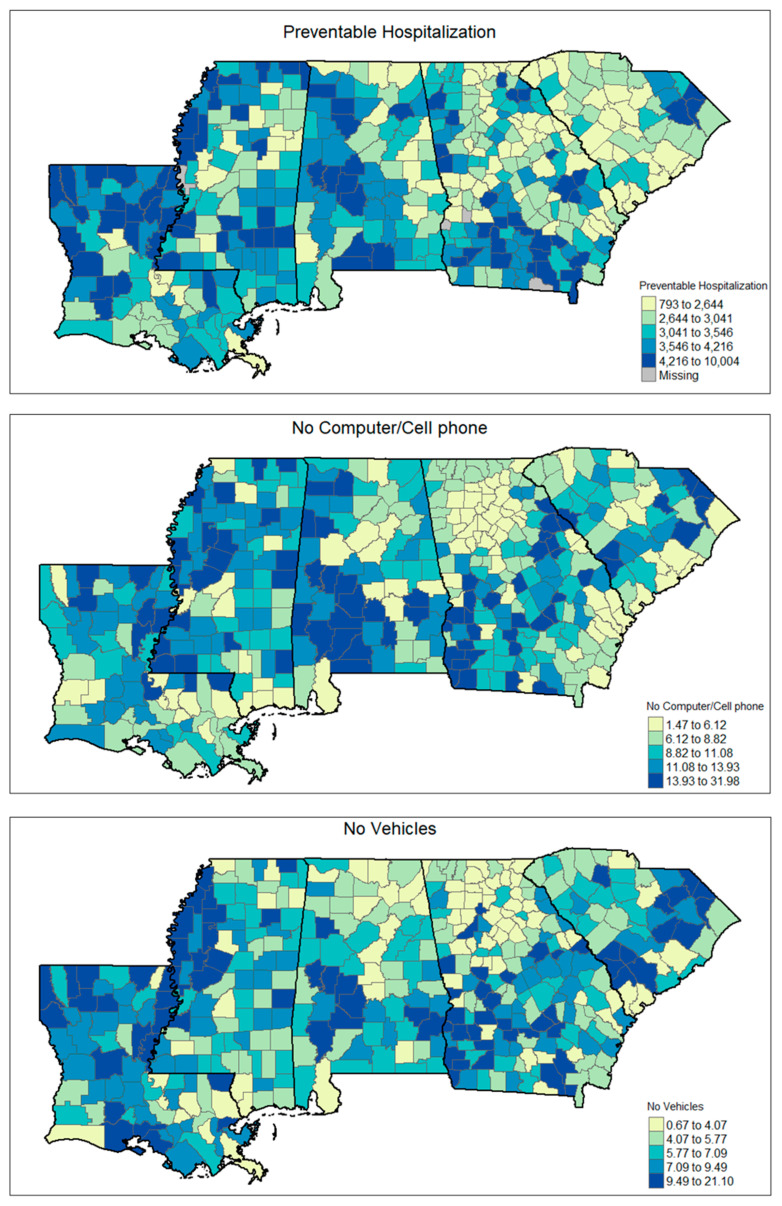

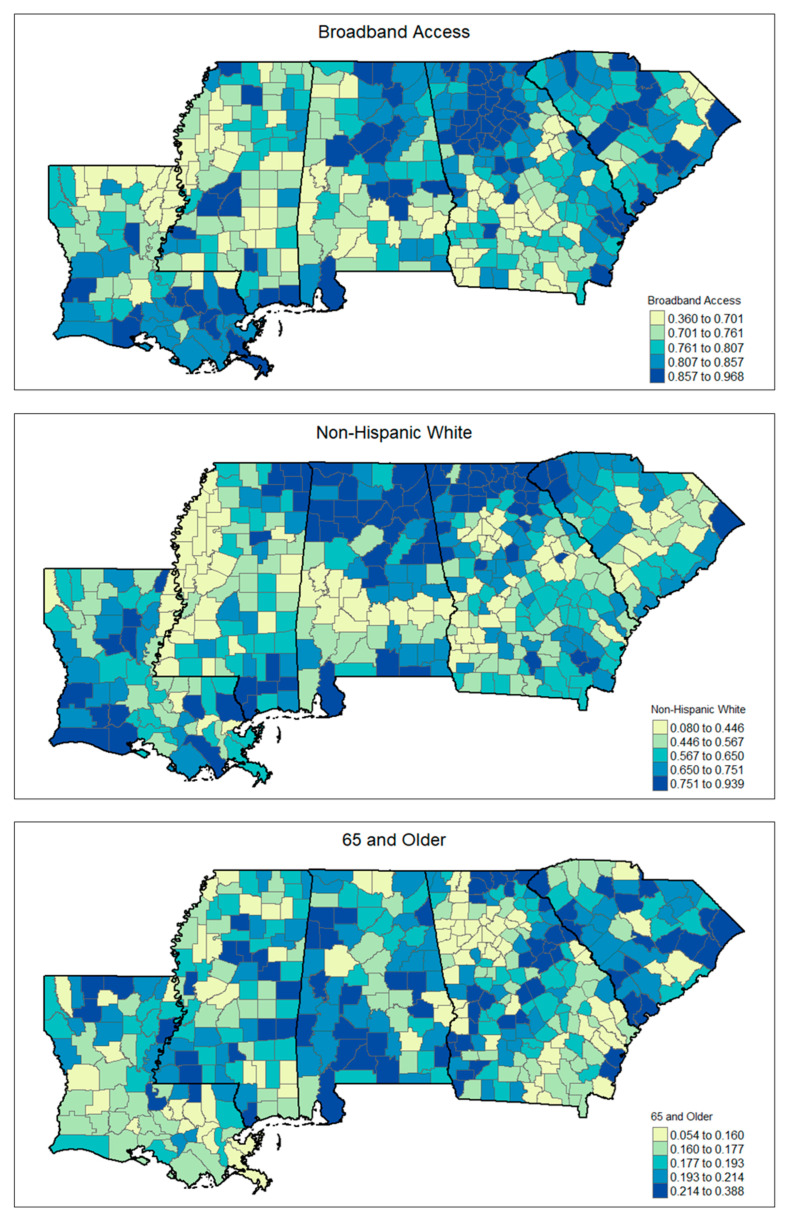

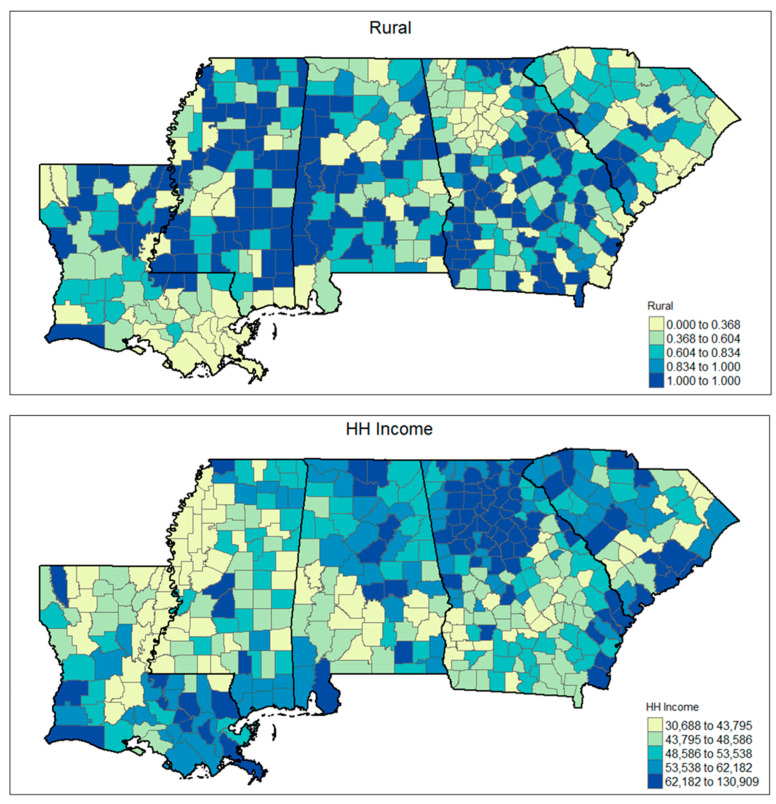
The spatial distribution of the study variables.

**Figure 2:**
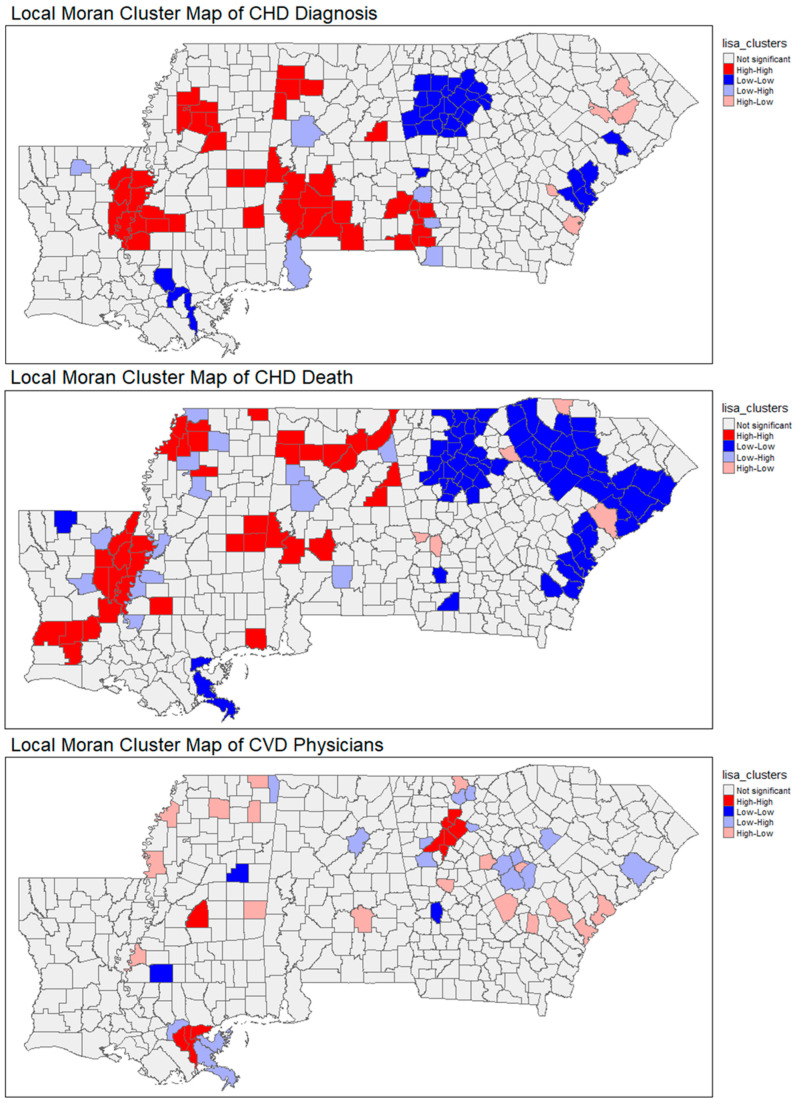

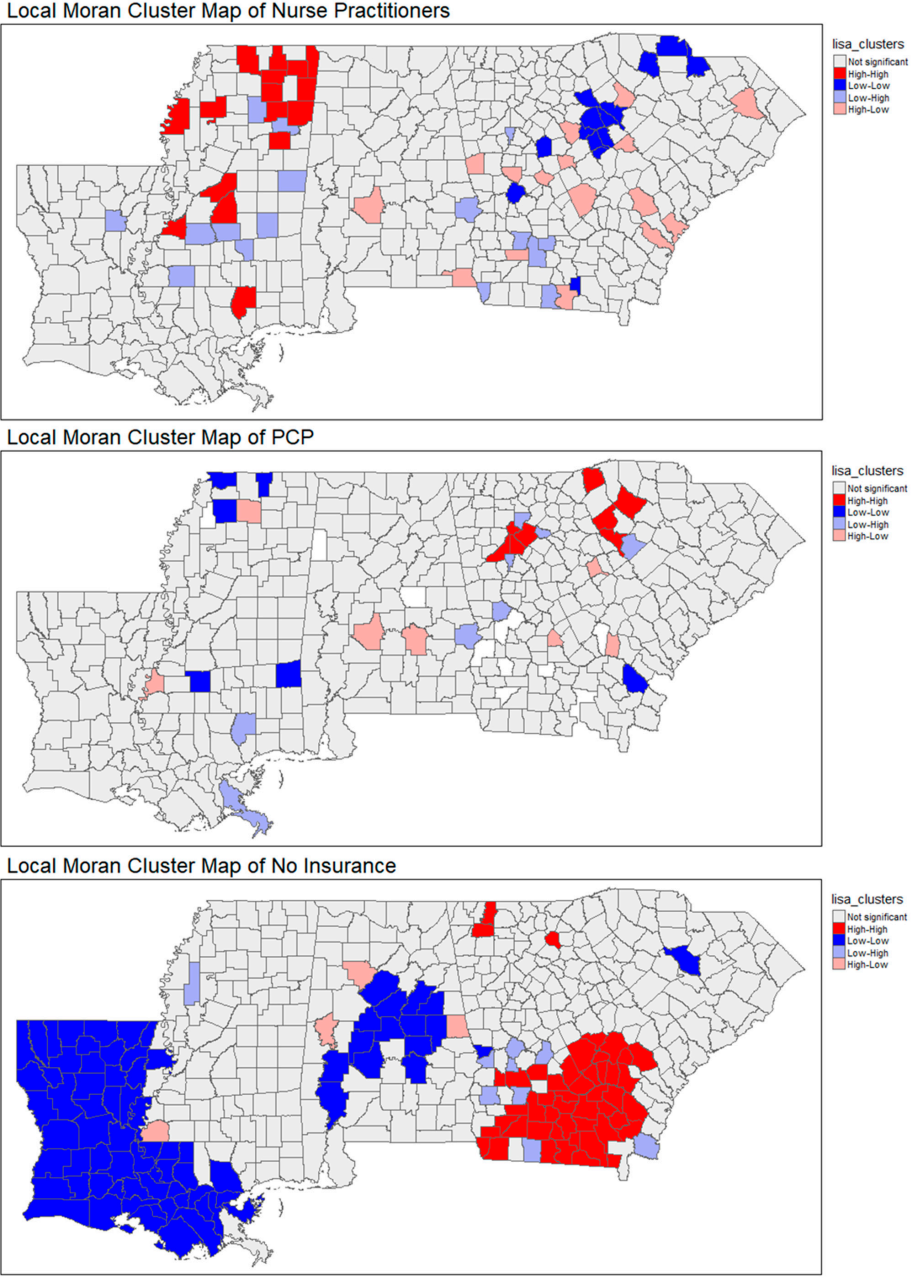

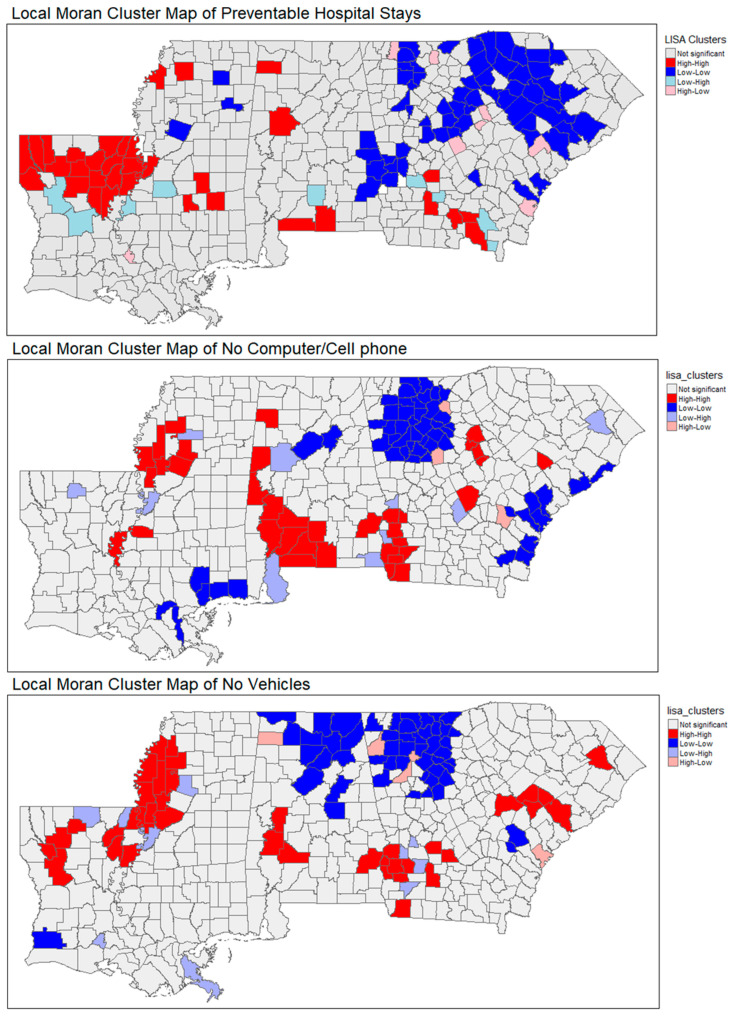

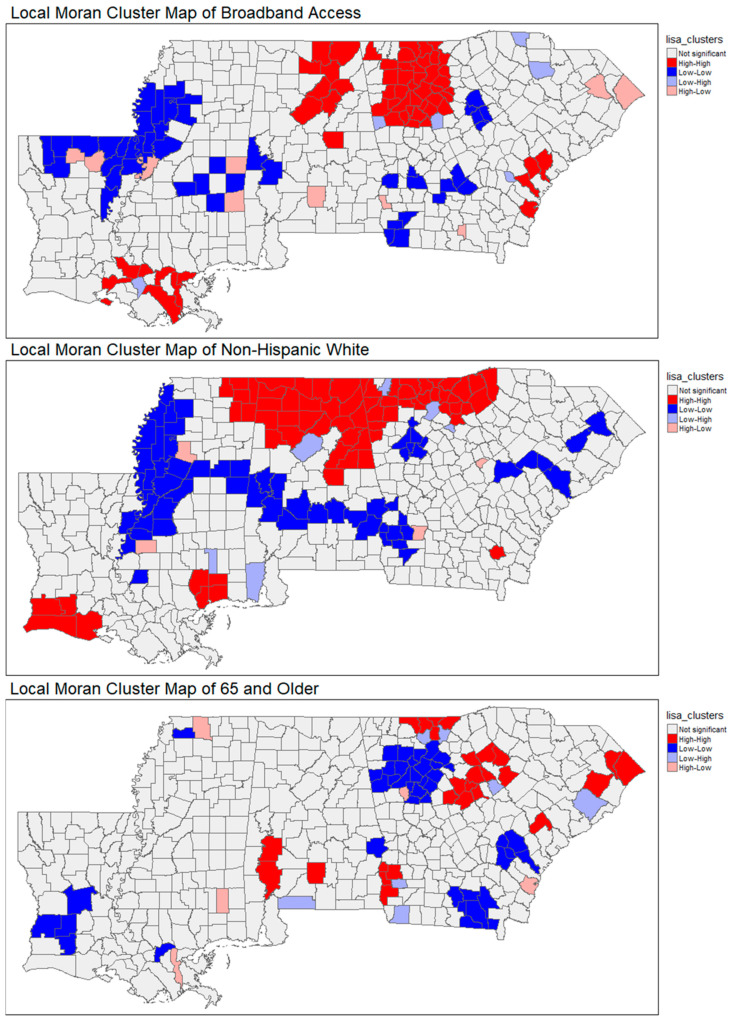

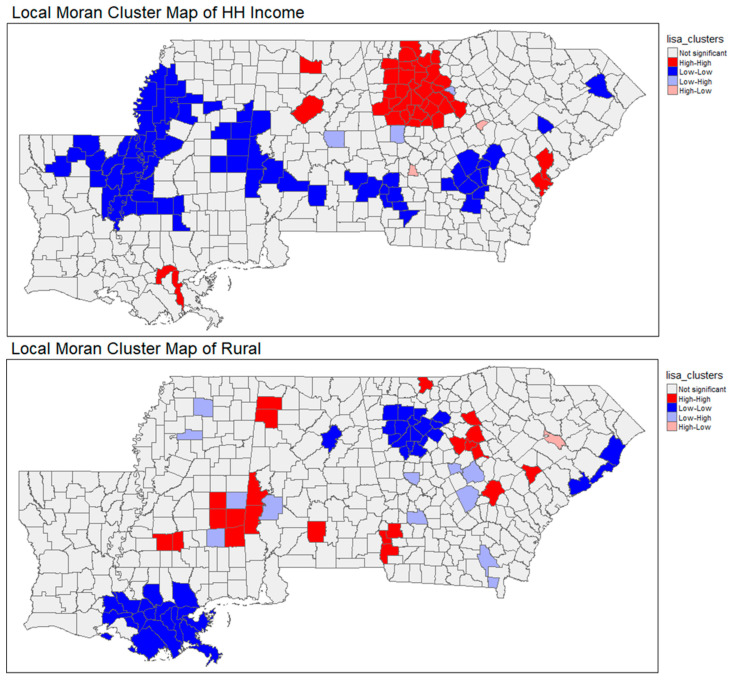
LISA maps of the study variables.

For CHD mortality, high–high clusters were observed in counties across the Mississippi Delta and parts of the western Alabama regions. Those counties are historically marked by socioeconomic disadvantages, healthcare workforce shortages, and persistent health disparities. Notably, these clusters aligned with counties having lower median household incomes, higher levels of uninsurance, and greater reliance on limited transportation infrastructure, such as households without access to a vehicle. The spatial mismatch between healthcare needs and healthcare availability was further illustrated by the presence of low–low clusters in more affluent suburban or metropolitan-adjacent counties, where provider availability and broadband access were higher. Please refer to Figure 1 and Figure 2 for a more detailed description of the spatial findings.

### 3.2. Multivariable Regression Results

To test this study’s three hypotheses derived from the Andersen healthcare utilization model, two separate linear regression models were estimated: one predicting CHD mortality and another predicting CHD prevalence. Variables were selected based on the theoretical framework, representing predisposing, enabling, and need-related factors. The multiple linear regression model predicting CHD death rates showed several significant indicators of healthcare accessibility and telemedicine readiness. A higher number of primary care physicians per county was significantly associated with lower CHD death rates (*B* = −54,430.00, 95% CI: −98,353.63 to −10,501.61, *p* = 0.015), supporting Hypothesis 3, which posited that greater access to healthcare providers would be protective. Similarly, households without access to a computer or cell phone, a key indicator of digital exclusion, were significantly associated with higher CHD mortality (*B* = 4.48, 95% CI: 0.93 to 8.02, *p* = 0.013), supporting Hypothesis 1.

Among the other variables, preventable hospital stays were also positively associated with CHD death (*B* = 0.02, 95% CI: 0.01 to 0.03, *p* < 0.001), potentially indicating system-level strain or a lack of effective chronic disease management. In contrast, broadband access was not significantly related to CHD mortality (*p* = 0.470), suggesting that broader digital infrastructure may have role in mortality than in earlier stages of disease prevention or diagnosis.

Age structure also played a role: counties with a higher percentage of older adults experienced significantly higher CHD mortality (*B* = 373.90, 95% CI: 119.13 to 628.72, *p* = 0.004), consistent with Hypothesis 2 regarding predisposing characteristics. Median household income was inversely associated with mortality (*B* = −0.003, 95% CI: −0.005 to −0.001, *p* = 0.000), further supporting Hypothesis 3 by highlighting the protective role of economic resources. The model explained 41% of the variance in CHD mortality (*R*^2^ = 0.41). The full results are presented in Table 3.

In the model predicting CHD prevalence, a similar pattern emerged. Counties with greater primary care physician availability had significantly lower diagnosis rates (*B* = −421,700.00, 95% CI: −777,671.30 to −65,729.06, *p* = 0.020), again supporting Hypothesis 3. Conversely, counties with higher rates of preventable hospitalizations (*B* = 0.13, 95% CI: 0.06 to 0.19, *p* < 0.001) and higher proportions of households without computers or cell phones (*B* = 39.33, 95% CI: 10.61 to 68.06, *p* = 0.007) had higher CHD prevalence rates, supporting Hypothesis 1.

Access-related barriers were also reflected in the finding that housing units without vehicle access were significantly associated with a higher prevalence (*B* = 33.88, 95% CI: 3.53 to 64.22, *p* = 0.029), suggesting that transportation limitations may hinder both preventive care and early diagnosis.

Demographic characteristics again played a role. Counties with a higher proportion of older adults (*B* = 17,980.00, 95% CI: 15,918.07 to 20,047.71, *p* = 0.0001) and those with more non-Hispanic White residents (*B* = 833.80, 95% CI: 378.53 to 1289.12, *p* = 0.0001) had significantly higher prevalence rates, consistent with Hypothesis 2. A higher median household income was associated with lower CHD prevalence (*B* = −0.03, 95% CI: −0.04 to −0.02, *p* = 0.0001), reinforcing the role of enabling resources outlined in Hypothesis 3. Please refer to Table 4 for a more detailed description of the findings.

## 4. Discussion

This study highlighted important geographic differences in CHD outcomes across the Deep South. The regression and spatial clustering analyses were structured to test three hypotheses derived from the Andersen healthcare utilization model, focusing on how enabling resources, predisposing characteristics, and access barriers relate to cardiovascular burden.

The findings were consistent with this study’s three guiding hypotheses. Specifically, the role of provider shortages and digital exclusion and less favorable CHD outcomes supports the hypothesis that limited enabling resources contribute to cardiovascular burden (Hypothesis 1). Similarly, demographic predictors such as the proportion of older adults were associated with worse outcomes, consistent with Hypothesis 2. Enabling factors such as provider availability and household income were protective, supporting Hypothesis 3. These findings align with the concept of the southern rural health penalty and reinforce concerns that systemic barriers continue to drive differences in cardiovascular health.

The spatial analysis of rurality, including both choropleth and the LISA cluster maps, highlights how high–high rural clusters, particularly in southern Louisiana and northeastern Alabama, are spatially aligned with high CHD burden. These patterns align with Hypotheses 1 and 2, illustrating how geographic isolation and demographic risk factors converge in counties with structural limitations. These clusters are geographically isolated and structurally constrained by low provider density and poor digital infrastructure. This reinforces the understanding that rurality is not simply a geographic classification, but a proxy for broader systemic disadvantage that manifests in diminished healthcare access, delayed diagnoses, and fragmented continuity of care. In these high-rural counties, health system underinvestment and long travel distances to providers present persistent barriers to timely intervention. The study findings indicate that broadband expansion alone is unlikely to improve health outcomes unless paired with parallel investments in the physical healthcare infrastructure. Highly rural communities may benefit most from hybrid solutions that combine telehealth access with in-person support from mobile clinics or community-based providers.

The multivariable analyses showed that provider availability measured by primary care physicians was significantly associated with lower CHD prevalence and mortality. This supports Hypothesis 3, which predicted that access-enabling factors would be protective against cardiovascular burden. This finding aligns with prior research that highlighted the lack of cardiologists and preventive care resources in rural southern counties [42]. As a majority of CHD outcomes are preventable, these shortages likely contribute to both underdiagnosis and unmanaged disease progression [43]. Additionally, residents lacking regular access to providers miss key preventive education, including guidance on nutrition and lifestyle modification [44,45].

Also, the study findings showed that digital exclusion measured by low household computer/mobile device access, is strongly associated with higher CHD mortality rates. This finding provides strong support for Hypothesis 1, suggesting that digital infrastructure is a key enabling resource necessary for disease prevention and management. These findings align with prior research demonstrating that broadband is a core enabler of healthcare access [20,21]. The National Digital Inclusion Alliance (NDIA) emphasizes that communities with poor digital infrastructure face combined health disparities, especially in the American South [46].

Broadband access was not a significant predictor of mortality. This might suggest that its role is strongest in prevention and early detection rather than acute disease management. In digitally underserved counties, the promise of telehealth remains largely unfulfilled. These findings support Hypothesis 1, suggesting that specific types of digital access (e.g., devices in the home) may be more directly influential than regional broadband coverage alone. Connectivity gaps restrict access to remote consultations, remote monitoring, and virtual follow-ups. This might exacerbate negative health outcomes where in-person care is already scarce [46]. These findings reinforce the growing recognition that digital access is a social determinant of health. To realize the full potential of telemedicine, particularly in the Deep South, public health strategies might prioritize digital inclusion alongside healthcare infrastructure expansion.

Importantly, our study identified counties that outperformed expectations despite facing structural disadvantages. These counties partially deviate from Hypothesis 1, suggesting that localized resilience, through unmeasured enabling factors, may mitigate expected health burdens even in structurally challenged counties. For instance, Washington County, Alabama and Jefferson Davis Parish, Louisiana both exhibit high rurality, low broadband access, and below-average provider density, yet reported lower-than-expected rates of CHD mortality. Similarly, Treutlen County, Georgia, while highly rural and economically disadvantaged, demonstrated better-than-average cardiovascular outcomes relative to the counties with similar risk profiles. These findings suggest that certain community-level factors or localized innovations may be helping to buffer the impact of structural barriers. Examples may include the presence of community health workers, strong social capital, mobile health outreach, local health partnerships, or targeted state-level funding that supports health promotion activities. Future studies might explore these potential enablers of resilience to inform more place-based intervention strategies. The policy implications derived from our results emphasize the need for multi-sector investments in rural health systems. Local governments could prioritize initiatives that co-locate digital infrastructure development with expanded healthcare access, such as subsidizing broadband in medically underserved areas while also supporting provider incentives to practice in rural settings. Health systems might consider hybrid telehealth models that incorporate both virtual care and in-person community outreach, especially in counties with a high CHD burden and limited connectivity. Furthermore, regionally coordinated care and investment in mobile preventive services could represent scalable policy solutions that build upon existing community strengths. Investigating the strategies these counties employ may offer insights into data-informed, place-based interventions.

It is important to highlight the study limitations. While the Andersen model provided a useful structure to organize variables, future studies may consider expanding the framework to capture resilience and social capital, which could help explain the better-than-expected outcomes observed in some counties. This study uses aggregate (county-level) data, which limits the ability to generalize findings to individual-level behavior or outcomes. This limitation reflects the well-documented risk of ecological fallacy (inferring individual-level relationships from group-level data) [47]. Therefore, while the findings provide insight into population-level patterns, they should not be interpreted as causal or predictive at the individual level. The generalizability of the findings is limited due to the study’s geographic focus on 418 counties in five Deep South states: Alabama, Georgia, Louisiana, Mississippi, and South Carolina. These states share specific structural, economic, and demographic profiles, including higher rates of poverty, systemic healthcare underinvestment, and digital infrastructure challenges. As such, the associations observed between provider availability, and cardiovascular outcomes may not hold in other rural regions of the U.S., such as Appalachia or the Mountain West, where contextual factors differ. Despite these limitations, this study highlights the need for multi-level, place-sensitive approaches to reduce CHD disparities in the Deep South. Investment in digital infrastructure might be matched with initiatives to support provider availability, reduce transportation barriers, and expand preventive health services. Policies that promote rural workforce development, such as telehealth training, rural residency programs, and loan forgiveness for service in high-need areas, could be considered.

## 5. Conclusions

This study highlighted that cardiovascular health outcomes in the Deep South are shaped by the geographic and structural factors that define rural life. The spatial clustering of high CHD burden aligns with the counties facing provider shortages, digital exclusion, and broader infrastructural challenges. While telehealth offers a promising tool to bridge access gaps, its potential remains limited in areas lacking the digital foundation needed for delivery. This study’s findings confirm the relevance of access-enabling and predisposing factors, as outlined in the Andersen framework, and highlight the importance of addressing multiple barriers simultaneously. At the same time, the identification of resilient counties that perform better than expected shows opportunities to learn from locally driven solutions that may be scalable across similarly underserved regions. To close the rural health gap in cardiovascular outcomes, a combined investment in both human and digital infrastructure is important.

## Figures and Tables

**Table 1:** Study variables.

Variable	Description	Role	Variable Type	Source
Heart Disease Prevalence	Coronary Heart Disease Prevalence by County, 2022, as of 2022 in Deep South.	Outcome	Continuous	PolicyMap [37]
Heart Disease Mortality	Coronary Heart Disease mortality by County, 2022, as of 2022 in Deep South.	Outcome	Continuous	Centers for Disease Control and Prevention (CDC) [38]
Cardiologist	Cardiovascular Disease Physicians per 1000 People by County, 2020, as of 2021 in Deep South.	Covariate	Continuous	PolicyMap [37]
Nurse Practitioner	Nurse Practitioners per 1000 People by County, 2020, as of 2022 in Deep South.	Covariate	Continuous	PolicyMap [37]
PCP	Percentage of Adults Reporting to Have a Personal Doctor or Healthcare Provider by County, 2010, as of 2018 in Deep South	Covariate	Continuous	PolicyMap [37]
Uninsured population	Estimated percentage of all people without health insurance, between 2019 and 2023.	Covariate	Continuous	United States Census Bureau [39]
Houses with no vehicles	Estimated percentage of housing units for which no vehicles are available in 2019–2023.	Covariate	Continuous	United States Census Bureau [39]
HH without any type of computer	Estimated percentage of households without any type of computer, between 2019 and 2023.	Covariate	Continuous	United States Census Bureau [39]
Broadband access	Percentage of households with broadband internet connection. The 2025 Annual Data Release used data from 2019 to 2023 for this measure.	Covariate	Continuous	United States Census Bureau [39]
% White	Percentage of Non-Hispanic White Population by County, 2022, as of 2018–2022 in Deep South.	Covariate	Continuous	United States Census Bureau [39]
% Rural	The percentage of population living in a census-defined rural area, 2020.	Covariate	Continuous	United States Census Bureau [39]
HH Income	Median household income, 2023.	Covariate	Continuous	United States Census Bureau [39]
% 65 and Older	Percent of all people 65 or older, between 2019 and 2023.	Covariate	Continuous	United States Census Bureau [39]

PCP = Personal Care Provider; HH = House Hold.

**Table 2:** Descriptive statistics of the study variables.

Variable	Mean	Standard Deviation	Units
Heart Disease Prevalence	8517	1450.30	Per 100,000 population
Heart Disease Mortality	446.95	98.70	Per 100,000 population
Cardiologist	0.02	0.01	Per 1000 population
Nurse Practitioner	0.95	0.61	Per 1000 population
PCP	0.04%	0.03%	Percent (%)
Uninsured Population	14.19%	3.39%	Percent (%)
Houses with No Vehicles	6.91%	3.27%	Percent (%)
HH Without Any Type of Computer	10.31%	4.78%	Percent (%)
Broadband Access	77.44%	9.33%	Percent (%)
% White	59.41%	17.88%	Percent (%)
% Rural	67.84%	31.20%	Percent (%)
HH Income	$54,114	$14,039	USD (Median Household Income)
% 65 and Older	18.92%	3.92%	Percent (%)

**Table 3:** Multiple linear regression predicting coronary heart disease death rates based on healthcare accessibility and telemedicine readiness indicators.

		95% CI			
	*B*	Lower Limit	Upper Limit	*p* Value	VIF
(Intercept)	430.20 **	243.55	616.83	0.000	
Cardiovascular Disease Physicians	−190.60	−440.34	59.09	0.134	1.86436
Nurse Practitioners	−32.76	−16.00	49.51	0.49	2.3033
Primary Care Physicians	−54,430.00 *	−98,353.63	−10,501.61	0.015	1.24876
No Insurance	2.78	−5.14	−0.41	0.21	2.38506
Preventable Hospital Stays	0.02 **	0.01	0.03	0.000	1.70738
Households without Computer/Cell phone	4.48 *	0.93	8.02	0.013	3.1081
Housing with No Vehicles	2.60	−1.15	6.34	0.173	1.7547
Broadband Access	61.22	−105.20	227.65	0.470	1.09798
% Non-Hispanic White	127.70	−71.48	183.85	0.100	3.41786
% 65 and Older raw value	373.90 **	628.72	119.13	0.004	3.79143
Median Household Income	−0.003 **	−0.001	−0.005	0.000	1.59585
% Rural	−0.10	−42.34	42.14	0.996	2.92088

Note: *p* < 0.05 (*); *p* < 0.01 (**); values indicate statistically significant results.

**Table 4:** Multiple linear regression predicting coronary heart disease rates based on healthcare accessibility and telemedicine readiness indicators.

		95% CI			
	*B*	Lower Limit	Upper Limit	*p* Value	VIF
(Intercept)	5725.00 *	4212.35	7237.34	0.0001	
Cardiovascular Disease Physicians	−1059.00	−3082.23	965.07	0.304	1.86436
Nurse Practitioners	99.47	−36.31	235.24	0.151	2.3033
Primary Care Physicians	−421,700.00 *	−77,7671.30	−65,729.06	0.020	1.24876
No Insurance	−3.53	−22.69	15.65	0.718	2.38506
Preventable Hospital Stays	0.13 *	0.06	0.19	0.000	1.70738
Households without Computer/Cell phone	39.33 *	10.61	68.06	0.007	3.1081
Housing with No Vehicles	33.88 *	3.53	64.22	0.029	1.7547
Broadband Access	−912.60	−2261.32	436.09	0.184	1.09798
% Non-Hispanic White	833.80 *	378.53	1289.12	0.0001	3.41786
% 65 and Older raw value	17,980.00 *	15,918.07	20,047.71	0.0001	3.79143
Median Household Income	−0.03 *	−0.04	−0.02	0.0001	1.59585
% Rural	476.90 *	134.59	819.20	0.006	2.92088

Note: *p* < 0.05 (*); values indicate statistically significant results.

## Data Availability

The data analyzed in this study are publicly available from multiple sources. County-level data on coronary heart disease prevalence and mortality were obtained from the Centers for Disease Control and Prevention (CDC) and PolicyMap. Socioeconomic and digital infrastructure indicators were sourced from the United States Census Bureau and County Health Rankings. All data used in this study are aggregated at the county level and are publicly accessible. Specific links and dataset descriptions are provided in the References and Methods Section of the manuscript.
